# Symbionts out of sync: Decoupled physiological responses are widespread and ecologically important in lichen associations

**DOI:** 10.1126/sciadv.ado2783

**Published:** 2024-06-14

**Authors:** Abigail R. Meyer, Natália M. Koch, Tami McDonald, Daniel E. Stanton

**Affiliations:** ^1^ Department of Ecology Evolution and Behavior, University of Minnesota, Saint Paul, MN 55108, USA.; ^2^ Department of Biology, Saint Catherine University, Saint Paul, MN 55105, USA.

## Abstract

A core vulnerability in symbioses is the need for coordination between the symbiotic partners, which are often assumed to be closely physiologically integrated. We critically re-examine this assumed integration between symbionts in lichen symbioses, recovering a long overlooked yet fundamental physiological asymmetry in carbon balance. We examine the physiological, ecological, and transcriptional basis of this asymmetry in the lichen *Evernia mesomorpha*. This carbon balance asymmetry depends on hydration source and aligns with climatic range limits. Differences in gene expression across the *E. mesomorpha* symbiosis suggest that the physiologies of the primary lichen symbionts are decoupled. Furthermore, we use gas exchange data to show that asymmetries in carbon balance are widespread and common across evolutionarily disparate lichen associations. Using carbon balance asymmetry as an example, we provide evidence for the wide-ranging importance of physiological asymmetries in symbioses.

## Teaser

Hydration-driven differences in symbiotic partner physiology can explain carbon balance and distributions of lichens.

## INTRODUCTION

When multiple organisms live together in symbioses, they must coordinate their metabolisms and physiologies to maintain the association (*1*). This coordination can break down if symbionts have asymmetric responses to stressors leading to the dissolution of symbioses (*2*), in particular in the face of climate disruptions (*3*). Despite recent evidence of stress-induced symbiotic breakdown (dysbiosis) in some associations such as corals (*4*), this phenomenon remains underexplored in many ecologically and evolutionarily important symbioses, including the first association recognized as symbiosis: lichens (*5*).

Lichen symbioses are a ubiquitous example of terrestrial symbiotic multispecies associations. Often used as an exemplar of symbiosis known for their association between fungi and photosynthetic algae and/or cyanobacteria, lichen symbioses are complex, routinely containing a suite of additional fungi and bacteria. While the exact role that each organism plays in lichen symbioses remains under examination (*6*–*9*), carbon balance in lichens has long been assumed to be maintained by carbohydrates fixed by the phototroph, which are transported to the fungus to fuel cellular respiration. Disruption of this carbon balance has been associated with bleaching and death of the lichen symbiosis (*10*). Less understood is the coordination of this carbon balance across natural cycles of wetting and drying.

Lichen symbioses lack the ability to maintain internal water status, quickly acclimatizing to external water conditions that bring them in and out of desiccation-induced dormancy. Lichen symbioses can use both liquid water and water vapor to rehydrate sufficiently to exit dormancy; however, because lichen symbioses are composed of multiple organisms, there may be differences in the activation thresholds of the component organisms. This could result in situations where one symbiont is physiologically active without the other. When exiting desiccation, some lichen thalli can regain full photosynthesis rates from exposure to water vapor alone (*11*), in some cases, within minutes to hours (*12*, *13*). Conversely, water vapor hydration activates low CO_2_ release rates as compared to liquid water hydration [shown, but not discussed in (*14*, *15*)]. Because carbon respiration is typically fungally dominated and carbon assimilation is uniquely algal, these observations suggest that water vapor hydration may differentially affect the primary symbionts, such that in some vapor conditions, only algae may be active (*16*).

These patterns point to potential asymmetries in physiological activities between components of the lichen symbiosis, forcing a critical re-evaluation of physiological integration in lichen symbioses. Some lichens have already been shown to have uncoordinated responses to longer scales of environmental variability, such as nutrient availability (*17*, *18*); however, asymmetric responses may be a regular and widespread attribute of many lichen symbioses. As part of a recent study of climate change effects on an iconic lichen symbiosis (*Evernia mesomorpha*) in high-humidity boreal forests (*10*), we encountered evidence of a marked carbon balance asymmetry between thalli rehydrated with water vapor versus liquid water at high temperatures. Despite strong evidence that this phenomenon may be an important part of lichen physiology (*19*), the ecological relevance of hydration with water vapor versus liquid water remains underexplored.

Through the present study, we examined the physiological, ecological and transcriptional basis of carbon balance asymmetry resulting from asymmetric activation of the lichen symbionts in the lichen *E. mesomorpha*. We also examine the occurrence of carbon balance asymmetry across multiple independent origins of lichen symbiosis. Using carbon balance asymmetry as an example, this work demonstrates the importance of examining the physiologies of the primary lichen-forming symbionts separately and provides further evidence for the wide-ranging importance of physiological asymmetries in symbioses.

## RESULTS

### Carbon balance asymmetry predicts ecological tolerances in an iconic boreal lichen

**Fig. 1.**
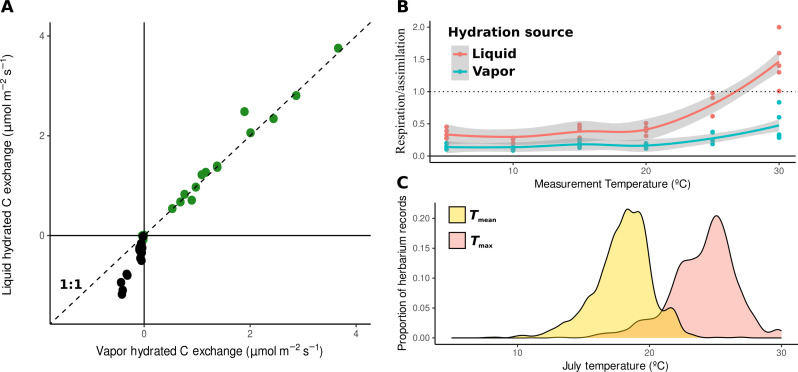
Physiological activity in *E. mesomorpha* thalli differs greatly between water sources, with increased carbon costs of liquid water at increasing temperatures. (**A**) Comparison of vapor and liquid hydrated rates of gross assimilation (green) and respiration (black) of *E. mesomorpha* thalli (*n* = 13 thalli). Dashed line indicates 1:1 rates of activity, showing no effect of hydration source on carbon fixation but greatly increased respiration in liquid. (**B**) Ratio of respiration to gross assimilation (*n* = 5 thalli/temperature level) as a function of temperature following vapor (light blue) and liquid (red) hydration, reflecting unsustainable carbon losses after liquid hydration above 20°C. (**C**) Distribution of mean (yellow) and maximum (red) July temperatures associated with North American records of *E. mesomorpha*, with range limits (maximum *T*_mean_) closely reflecting hydration-driven physiological limits.

To determine differences in carbon balance between thalli hydrated with water vapor versus liquid water, we conducted gas exchange measurements of *E. mesomorpha* thalli to quantify carbon assimilation (in the light) and respiration (in the dark) across six temperature levels (5°, 10°, 15°, 20°, 25°, and 30°C). In the water vapor hydration condition, photosynthetic activity in *E. mesomorpha* thalli activated rapidly, reaching high levels within 1 to 2 hours (fig. S1). Maximum gross carbon assimilation rates after hydration with water vapor were nearly identical to those obtained from liquid water hydration (slope = 1.03, *r*^2^ = 0.976, *F*_1,15_ = 652, and *P* < 0.001; Fig. 1A, green data points). In contrast, carbon respiration rates were approximately double in liquid water compared to water vapor–hydrated thalli (slope = 2.13, *r*^2^ = 0.885, *F*_1,15_ = 124, and *P* < 0.001; Fig. 1A, black data point). Light compensation points, representing the light intensity required for carbon assimilation to offset carbon respiration, were significantly higher in liquid water hydration conditions (fig. S2), and became ecologically unsustainable above 25°C, with some thalli never reaching positive carbon balance when hydrated with liquid water. The relative carbon cost of respiration increased sharply with temperatures above ~20°C, and positive carbon balance at 30°C was only possible when hydrated with water vapor (Fig. 1B). This temperature threshold for carbon balance coincides closely with the thermal range limits of *E. mesomorpha* in North America (Fig. 1C), suggesting that extreme carbon costs associated with summer rain events could be a key climatic stressor. The alignment of these physiological thresholds with natural climatic range limits points to the possibility of mechanistic predictions for geographic distributions and climate responses, combining new (and old) physiological data with greatly improving distribution (*20*) and climate (*21*) mapping.

Our findings demonstrate the ability for algae associated with *E. mesomorpha* to achieve maximum carbon assimilation from water vapor hydration alone. This suggests that the differences in thallus respiration rates when thalli are rehydrated with water vapor versus liquid water are primarily attributable to heterotrophic components of the symbiosis, which may be less active in vapor. This apparent carbon balance asymmetry is amplified at high temperatures, such that positive carbon balance becomes impossible under liquid water hydration conditions. Furthermore, extended periods of high humidity and temperature do occur frequently at a representative field site (fig. S1), reinforcing the ecological importance of water vapor use and the associated carbon balance asymmetry in the range limits of *E. mesomorpha* (Fig. 1, B and C).

### Metatranscriptomics reveal decoupled physiologies of the component organisms of *E. mesomorpha*

**Fig. 2.**
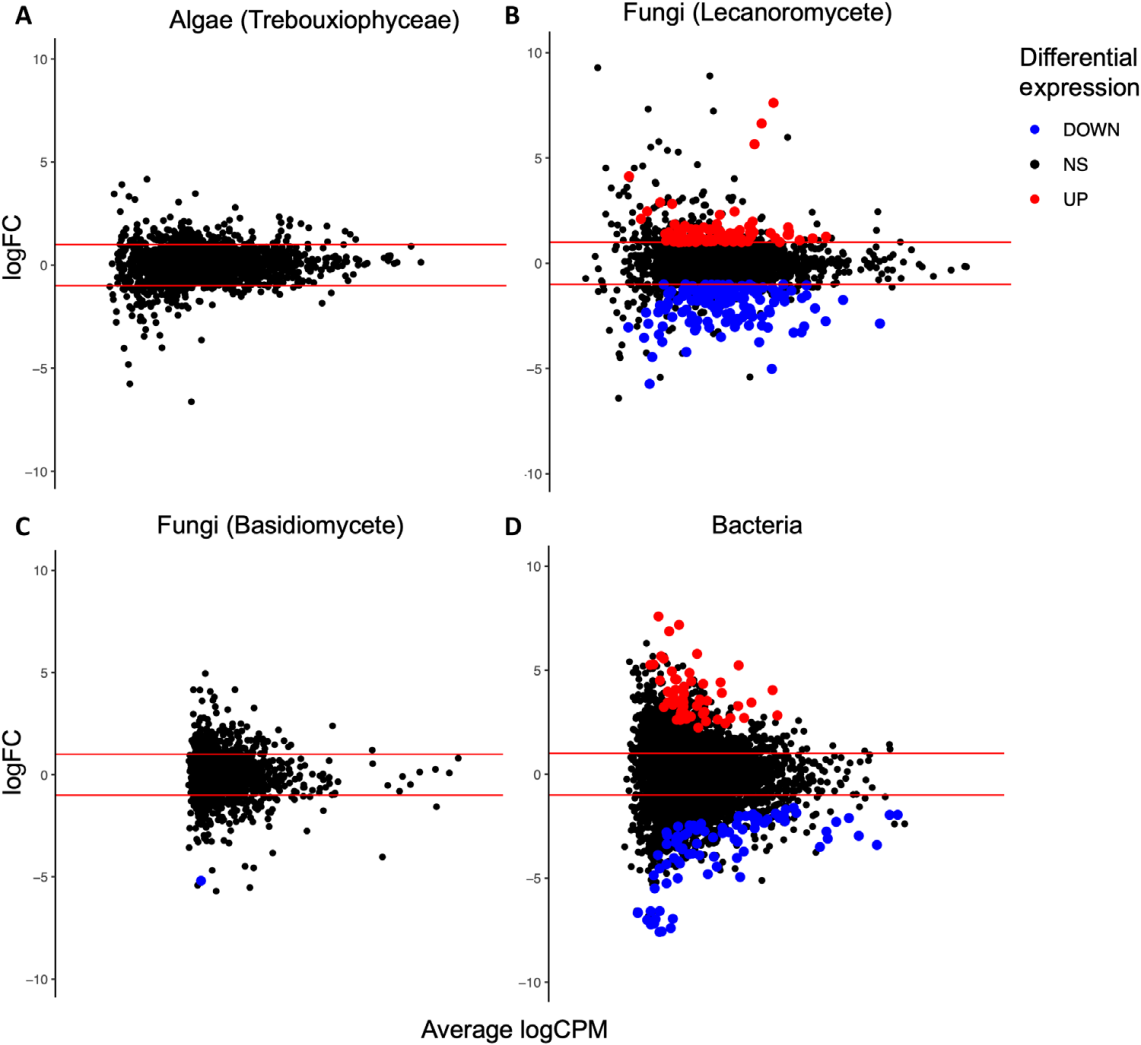
Metatranscriptomics shows the components of the *E. mesomorpha* lichen symbiosis respond differently to hydration sources. Panels show log2 fold change (logFC) of gene expression between water vapor and liquid water hydrated *E. mesomorpha* thalli (*n* = 5 thalli/condition) plotted against the average log counts per million (logCPM) transcripts for the (**A**) lichen-forming algae, (**B**) lichen-forming fungus, (**C**) other associated basidiomycete fungi, and (**D**) thallus-inhabiting bacteria. Blue and red points indicate significant differential expression (false discovery rate < 0.05 and FC > |2|).

To test whether the observed carbon balance asymmetry in *E. mesomorpha* was due to a unique response of the lichen-forming fungus to hydration source, we sequenced the metatranscriptomes of dry, water vapor–, and liquid water–hydrated *E. mesomorpha* thalli (n = 5 per condition). Transcription did not change for the lichen-forming alga when comparing liquid water versus water vapor–hydrated thalli (Fig. 2A), supporting previous observations that water vapor is sufficient to activate photosynthesis for many lichen-forming algae (*13*). For the lichen-forming fungus, 85 genes were significantly upregulated, and 147 genes were significantly downregulated (Fig. 2B), suggesting that lichen-forming fungi undergo additional physiological changes when hydrated with liquid water as compared to water vapor. This change in gene expression for the lichen-forming fungus in liquid water hydrated thalli is not accompanied by changes in gene expression for the lichen-forming algae, suggesting the lichen-forming algae may not be responding to physiological shifts that occur for the lichen-forming fungus upon hydration with liquid water. This suggests that the physiologies of the primary lichen symbionts are, at least in part, decoupled from one another.

Differences in gene expression across the component organisms in the *E. mesomorpha* symbiosis suggest that the lichen-forming fungi and lichen-associated bacteria may have a unique carbon respiration response under liquid water hydration conditions. Gene ontology (GO) enrichment analysis of the 230 differentially expressed lichen-forming fungal genes (DEGs) between water vapor– and liquid water–hydrated thalli did not show enrichment of biological processes linked to CO_2_ respiration (table S1). However, two of three 2-oxoglutarate dehydrogenase genes (GO: 000610) were differentially expressed, and 2-oxoglutarate dehydrogenase has been indicated as a gene marker for fungal respiration (*22*). Contrary to our predictions, hierarchical clustering and visualization of all tricarboxylic acid cycle and sugar transporter lichen-forming fungus genes did not show shifts in gene expression in response to hydration with liquid water as compared to water vapor (figs. S3 and S4). We also checked for shifts in gene expression in other organisms that are commonly found in lichen thalli (*8*) that may explain the increase in CO_2_ respiration in liquid water hydrated thalli. Lichen-associated basidiomycetes (Fig. 2C) repeated the pattern observed with the lichen-forming alga, showing no significant transcriptional changes between water vapor and liquid water hydrated thalli. Lichen-associated bacteria showed differences in gene expression (Fig. 2D). GO enrichment analysis of lichen-associated bacteria DEGs between water vapor and liquid water hydrated thalli indicated the ethanol oxidation GO term (GO: 0006069) to be enriched (*P* = 0.0469), which could result from a shift to anaerobic respiration, i.e., ethanol fermentation, which would cause an increase in CO_2_ production (table S2). That these fungal and bacterial changes are not paired with any significant transcriptional changes in the lichen-forming alga is clear evidence of the physiological decoupling driving the carbon balance asymmetry.

### Carbon balance asymmetries in lichens are widespread and common

**Fig. 3.**
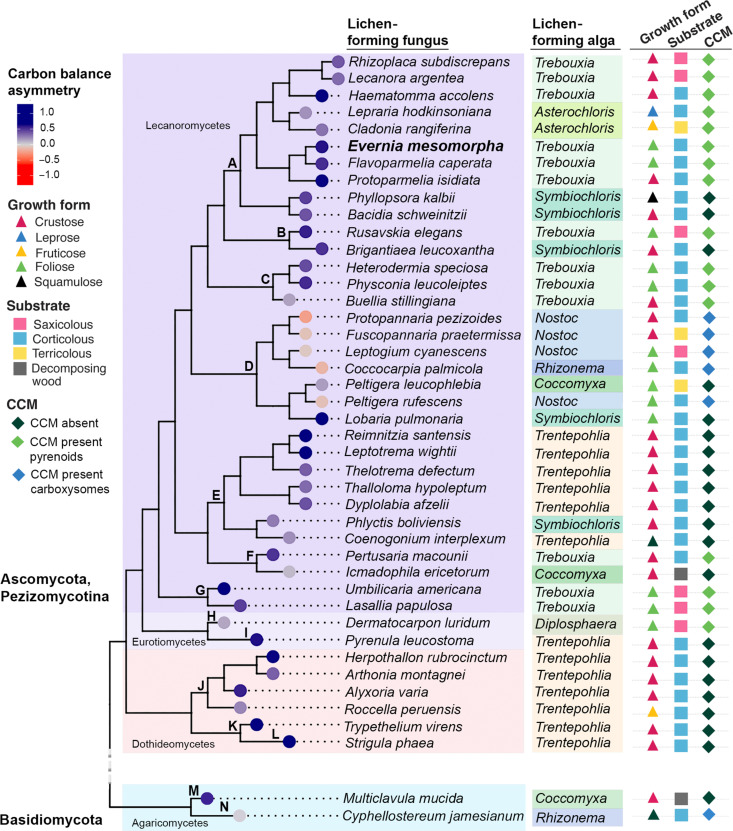
Carbon-balance asymmetry is widespread across lichen symbiosis origins and ecologies. Cladogram showing the phylogenetic relationships of the genera of the lichen-forming fungi tested in this study, their carbon balance asymmetry (branch tips), genera of lichen-forming algae, and lichen functional traits (symbols in the right). Positive values of carbon balance asymmetry are shown in shades of blue and negative values, in shades of red. Functional traits are represented by growth form (triangles), substrate (squares) and algal physiology (CCM, diamonds). Orders: A, Lecanorales; B, Teloschistales; C, Caliciales; D, Peltigerales; E, Ostropales; F, Pertusariales; G, Umbillicariales; H, Verrucariales; I, Pyrenulales; J, Arthoniales; K, Trypetheliales; L, Strigulales; M, Cantharellales; N, Agaricales.

While the *E. mesomorpha* lichen association is a “classic” macrolichen, it may not be representative of the polyphyletic diversity of lichen-forming fungi and algae. Taxonomic and geographic biases in sampling have constrained the current state of understanding of lichen physiology (*16*), and published studies of water vapor hydrated gas exchange are no exception even if they suggest that carbon balance asymmetry is widespread phenomenon (table S5). To overcome this, we collected thalli from a range of habitats, from boreal to mediterranean and subtropical, in the United States to examine the frequency of carbon balance asymmetries across lineages, growth forms, habitats, and algal physiology (table S6). A total of 41 lichen associations were studied, representing seven independent fungal origins of lichenization (*23*) and six lichen-forming algal/cyanobacterial families (Fig. 3). To summarize and compare physiological asymmetries, we calculated an index of carbon balance asymmetry, reflecting the relative activation of carbon assimilation (algae/cyanobacteria only) and respiration (all symbionts) in water vapor. Positive index values reflect asymmetries favoring carbon assimilation in water vapor, while negative values indicate greater amounts of respiration than assimilation in water vapor. We generated a cladogram based on recent publications (*23*, *24*) and calculated the carbon balance asymmetry of each taxon based on physiological measurements.

Of the 43 lichen associations measured, 38 had positive asymmetry values, including members of all growth forms, growth substrates, and carbon physiologies (Fig. 3). This demonstrates that the strong carbon balance benefit of water vapor hydration observed in *E. mesomorpha* thalli is a widespread phenomenon in lichens, appearing across a diverse range of lichen-forming fungi and algae (Fig. 3), including members of all 14 fungal orders examined, all green algal lineages, and even, albeit weakly, one cyanobacterial lineage (*Rhizonema* in *Cyphellostereum*).

Algal physiology, particularly as related to carbon-concentrating mechanisms (CCMs), is strongly associated with environmental distributions of lichen associations (*25*) and emerges as the strongest predictor of carbon assimilation from water vapor hydration. One monophyletic clade (D = Peltigerales) contained the only negative asymmetry index values. All of the negative values in this clade are from lichen-forming fungi associated with cyanobacteria, which have carboxysomes as their CCM. The green algal lichens tested from this clade (*Lobaria pulmonaria* and *Peltigera leucophlebia*) showed positive values. High levels of respiration activity in water vapor were rare (2 of 43 lichen associations) and may be associated with ecology, such as lichen associations from very humid (e.g., *Arthonia montagnei*) or high-salinity microhabitats (e.g., *Roccella peruensis*). Neither growth form nor substrate emerged as direct predictors of carbon balance asymmetry. Despite these interesting exceptions, carbon balance asymmetry is an unexpectedly widespread and common phenomenon across evolutionarily disparate lichen associations.

## DISCUSSION

Our results recover a long-overlooked physiological asymmetry at the heart of lichen function, with major implications for both our understanding of this iconic symbiosis and predictions of their climate responses. The activation of carbon assimilation and respiration of *E. mesomorpha* thalli is asymmetric and dependent on type of hydration with consequences for carbon balance in lichens. Hydration with liquid water is associated with greatly increased carbon losses through respiration, which reach unsustainable levels at ecologically realistic high temperatures (25°C). This threshold for viable carbon balance is very close to the summer maximum temperatures in the geographic range of *E. mesomorpha* (Fig. 1C), such that increasing summer wetting could drive widespread mortality. These results suggest that carbon balance asymmetry between symbionts would therefore seem to be a key predictor of environmental tolerance in lichen symbioses.

Our metatranscriptomes confirm that hydration with liquid water induces changes in the physiology of the primary lichen-forming fungus that are not matched in the lichen-forming alga (Fig. 2, A and B). This change in gene expression on the part of the lichen-forming fungus does not appear to induce shifts in gene expression for the lichen-forming alga, suggesting some degree of decoupling in physiological responses to both hydration and physiological shifts occurring in the other components of the symbiosis. The decoupling of physiological responses has also been shown between different cyanobacteria in the cyanolichen *Lichina pygmea* (*26*). Metatranscriptomic analyses across a diversity of lichen symbioses will likely continue to show physiological asymmetries in response to different environmental conditions.

The degree to which the observed shifts in gene expression between water vapor– and liquid water–hydrated thalli can be attributed to biological processes connected to CO_2_ respiration is limited. Gene expression shifts associated with respiration in the lichen-forming fungus and the potential for ethanol fermentation in the lichen-associated bacteria both stand out as potential pathways that could explain increased CO_2_ production in liquid water–hydrated thalli. Ethanol fermentation due to O_2_ diffusion limitation associated with high hydration conditions has been demonstrated in some lichen associations (*27*), and it could be a critical biological process for both lichen-forming fungi and lichen-associated bacteria when hydrated with liquid water. The degree to which other microbial partners are integrated within the carbon economy of lichen symbioses remains to be determined, making the identification of the source of increased CO_2_ respiration under liquid water hydration conditions imperative for predicting lichen response to climate change.

The phenomenon of carbon balance asymmetry is very widespread (but not universal) across lichen-forming lineages, growth forms, and habitats. A critical re-examination of past studies (*14*) reveals it to have been documented, yet its biological and ecological relevance was largely unrecognized [e.g. (*19*)]. Although the specific thresholds will differ across taxa (and perhaps populations), the increased imbalance between respiration and carbon assimilation rates with increasing temperature is universal, and so, most likely, is the physiological advantage of water vapor hydration. At elevated temperatures, water vapor serves as a hydration source associated with reduced carbon losses and is thus most likely a key element of lichen thallus ecophysiology in humid tropical and subtropical climates, as well as temperate climate summers. Furthermore, chlorophyll fluorescence measurements demonstrate that only short periods (1 to 2 hours) of high humidity are required for *E. mesomorpha* thalli to use water vapor to activate carbon fixation. Similarly, rapid use of water vapor has also been reported for a number of other lichen associations (*13*). *E. mesomorpha* frequently experiences periods of sufficient humidity for water vapor hydration (fig. S1), and extended periods of high humidity and temperature are common in many ecosystems.

The contrasting carbon balance between thalli hydrated with water vapor versus liquid water also points to a likely mechanism for lichen vulnerability to climate events. Wetting events during periods of high temperature, such as summer rains, have the potential to cause irrecoverable carbon imbalances. Similar phenomena have been reported in desert biocrusts, where increased frequency of small summer rains led to dramatic mortality (*28*). This points to an urgent need for better physiological grounding of lichen distribution models: Any forecasting of climate change impacts on lichens will need to incorporate this physiological asymmetry and the ecological importance of humidity as separate from rainfall.

None of this will be complete without more detailed understanding of the mechanisms generating the physiological asymmetries we report here. Determining both the organisms and biochemical pathways responsible for increased carbon losses under liquid water hydration is critical for creating a model of lichen carbon balance under different climatic conditions. If the increase in CO_2_ respiration in liquid water hydrated lichens is from microorganisms not integrated into the carbon economy of lichen symbioses (i.e., the lichen thallus as habitat as opposed to source of carbon), current tools to measure respiration in lichen symbioses are inadequate to capture the carbon balance of lichen symbioses. Although the reduced carbon costs of water vapor hydration may seem advantageous, what are the long term impacts for each of the component organisms? Many lowland tropical lichens are highly hydrophobic (*29*), while other lichens (e.g., *Lepraria*, calicioids) occur in sheltered microhabitats where they may never experience liquid water; these can provide insights into long-term adaptations to liquid water-free conditions. Furthermore, some lichen thalli contain the presence of multiple genotypes (or even species) of alga and fungus (*10*, *30*, *31*), which could influence asymmetries and create mechanisms for acclimation through turnover.

Ecophysiological data and molecular data together suggest the common portrayal of the lichen symbiosis as a highly integrated physiological unit may be inaccurate. Physiological asymmetries like the one outlined here challenge our understanding of the integration of symbioses and may be a key driver of the breakdown of these intimate interactions, in particular in the face of climate change. Similar nuance is needed in the study of other closely integrated symbioses, as in recent detailed studies of coral bleaching [e.g., (*4*)], especially where physiological integration has been assumed. Recent advances in mutualism theory (*32*) offer some guidance, but the unexpected gaps in knowledge revealed by the present study (e.g., which organisms and processes are even responsible for CO_2_ production in hydrated lichens) highlight the need for this theory to be paired with close examinations of symbiosis biology.

## MATERIALS AND METHODS

### Ecophysiology, *E. mesomorpha*

Fresh thalli (0.1 to 0.3 g) of *E. mesomorpha* Nyl. were collected from a *Picea mariana*–dominated bog at Cedar Creek Ecosystem Reserve, Anoka, Minnesota (N 45.4233, W 93.1902) in September 2022. Before measurement, thalli were thawed on the lab bench [25°C, ~50% relative humidity (RH)] until room temperature and air dry. To achieve vapor hydration, thalli were placed in sealed containers (~700 cm^3^) over distilled water for 12 hours at low light (<15 μmol m^−2^ s^−1^). These conditions achieve complete equilibration with humid air within hours (*13*) (fig. S1) and mimic natural nocturnal conditions. Assimilation and respiration rates were measured using a portable Infra-Red Gas Analyzer (LI-6800, LI-COR, Lincoln, NE, USA) equipped with an Aquatic Chamber (6800-18; internal volume of 20 cm^3^) that enables measurements under very high humidity conditions. High humidity in the measurement space is essential to minimize water loss from thalli during the course of measurements. Measurements were conducted at six temperature levels (5°, 10°, 15°, 20°, 25°, and 30°C) in a controlled temperature room. Chamber conditions were RH target 95% (realized RH ~91%), 500 μmol/s flow rate, and reference CO_2_ of 400 parts per million (ppm). Light response curves consisting of a 4- to 5-min stabilization stage followed by 10 repeated measurements at 2-s intervals were constructed from the following sequence of programmed photosynthetically active radiation (PAR) levels: 0, 1000, 2000, 2500, 1000, 500, 250, 100, and 50 μmol m^−2^ s^−1^. Because of light attenuation by the chamber walls, realized PAR values were as follows: 0, 544, 815, 1086, 1357, 544, 273, 136, 55, and 27 μmol m^−2^ s^−1^. Following vapor hydrated measurements, thalli were brought to maximal internal liquid hydration using standardized protocols (*10*) and remeasured following the same instrumental settings as above.

To determine the amount of time spent at high humidities that is required to achieve maximum photosynthetic efficiency (*F*_v_/*F*_m_), chlorophyll fluorescence measurements were performed using a red-light IMAGING-PAM m-series chlorophyll fluorometer (Walz, Effeltrich, Germany). The *E. mesomorpha* thallus was placed in a saturating humidity chamber and a saturating pulse was applied every 10 min for 8.5 hours to record *F*_v_/*F*_m_, which was automatically calculated in ImagingWinGigE version 2.56p.

### Bioclimatic range of *E. mesomorpha*

Georeferenced herbarium records of *E. mesomorpha* from USA and Canada were retrieved from the Consortium of North American Lichen Herbaria (*20*), providing 3600 records. For each record, we retrieved July mean and maximum temperature for 1970–2000 using WorldClim 2.1 climate data at 2.5-min resolution (*21*), using R package terra (*33*).

### Microclimate data for *E. mesomorpha*

We characterized the microclimatic environment typically experienced by *E. mesomorpha* using climate data from a representative site. The Spruce and Peatland Responses Under Changing Environments (SPRUCE) project, situated in Marcell Experimental Forest (near Grand Rapids, MN, USA), has high-resolution (30 min) climate data (*34*) for a black spruce (*P. mariana*) and *Sphagnum* bog in which *E. mesomorpha* is a dominant epiphytic lichen. While much of the experiment focuses on effects of warming and CO_2_, control plots are also instrumented. We retrieved air temperature and RH data at 1 m from a control plot (WEW PLOT_07) for the four most recent complete years (2018–2021). These data were used to calculate the daily hours of high humidity conditions (RH > 95%). Because the physiological importance of carbon balance asymmetry is greatest at high temperatures, we further subset the data to only include those periods where air temperature exceeded 20°C.

### Molecular

*E. mesomorpha* thalli were collected from Cedar Creek Ecosystem Science Reserve. Thalli were removed from *P. mariana* and *Larix laricina* branches with gloved hands and immediately brought back to the laboratory where they were cleaned of non-lichen debris (bark, needles, invertebrates, etc.), weighed, and temporarily stored in −20°C until RNA extractions the following day.

Lichen thalli were placed in one of three treatments for RNA extraction: dry, water vapor hydrated, and liquid water hydrated. The dry treatment consisted of five lichen thalli that were not hydrated and RNA extractions were performed in the dry state. The water vapor–hydrated treatment consisted of five lichen thalli set in a closed container at saturating humidity for 16 hours before RNA extraction. The liquid water treatment was similarly water vapor hydrated, but before RNA extraction, the five lichen thalli were hydrated with liquid DI H_2_O following the protocol described in (*10*) to reach internal water holding capacity. During the water vapor hydration stage, all thalli, including the dry condition, sat under a grow light, starting with 8 hours of dark followed by 8 hours of light.

RNA extractions were performed over the course of 3 days, using an RNeasy Plant Mini Kit extraction protocol with three modifications. (i) The initial buffer volume was increased to 1500-μl buffer RLC per 100-mg dry weight of lichen tissue and lichen tissue was ground directly in the lysis buffer with a mortar and pestle. (ii) Before moving on to the washing steps of the RNeasy Plant Mini Kit protocol (steps 6 to 8), a deoxyribonuclease (DNAse) step was performed following the protocol outlined in (*35*). After binding the extracted RNA/DNA to the pink RNeasy Mini spin column, 60 ml of DNase solution [52 μl of ribonuclease (RNase)–free water, 6 μl 10× DNase buffer Promega M198A, 2 μl RQ1 RNase-free DNase Promega M610A] was added to the column and incubated at 37°C for 15 min. Subsequently, 300 μl of a 1:1 solution composed of RNeasy Plant Mini Kit buffer RLT and 100% ethanol was added to the column and incubated at room temperature for 5 min. Last, the lysate was centrifuged at max speed for 15 s and the flow through containing sheared DNA was discarded and the RNeasy Plant Mini kit protocol was continued at step six. (iii) After an initial elution with 30 μl of RNase-free water, the eluate was added again to the column for a second elution to increase RNA concentration.

Extracted RNA was immediately put on ice and RNA concentration and 260/280 and 260/230 ratios were measured on a spectrophotometer 0 (NanoPhotometer NP80, Implen, Munich, Germany). After RNA quantification, samples were stored at −20°C until all extractions were completed. Before RNA sequencing samples were quantified again on an Agilent BioAnalyzer (Agilent Technologies, Santa Clara, CA, USA) at the University of Minnesota Genomics Center and determined to have an average RNA integrity value of 8.

RNA library prep and sequencing was performed by the University of Minnesota Genomics Center. RNA was mRNA purified with the stranded total RNA kit and sequenced on the NovaSeq 6000, 150 PE flow cell with a sequencing depth of ~58 million reads per sample.

### Computational

Before transcriptome assembly, rRNA reads were removed with RiboDetector (ribodetector_cpu -t 20 -l 150 -e rrna --chunk_size 1000) (*36*). Filtered, untrimmed reads (table S3) were then de novo assembled with Trinity (*37*). Assembly statistics are included in table S4. The Trinity utility align_and_estimate_abundance.pl with the -est-method Salmon was used to align each sample file to the combined Trinity assembly for estimating transcript abundance and creating an expression matrix.

To identify which symbiont each transcript originated from, open reading frames were computed from the full Trinity assembly using the Transdecoder plugin (TransDecoder.LongOrfs), and coding regions were subsequently predicted (TransDecoder.Predict). Predicted coding regions were then blastp searched against the National Center for Biotechnology Information nonredundant (nr) database using the blastp algorithm with the addition of the staxid output option to include taxonomic identifiers. Following (*38*) methods for assigning taxonomy to each transcript from blast results, the longest splicing isoform per transcript was identified and the best *E* value blast result over that isoform was used as the taxonomic assignment for that gene. If multiple blast results remained, only the first instance was kept. This isoform’s blast result was then used as the taxonomic identifier for the entire gene. The Taxonomizr package (*39*) was used to identify taxonomy from staxid. Of the 917,783 genes in the entire transcriptome assembly, taxonomy was able to be assigned for 236,076 genes, and 681,707 genes were unassigned and dropped from further analysis. Genes in the class Trebouxiophyceae were assigned to the lichen-forming algae, genes in the class Lecanoromycete were assigned to the primary mycobiont, genes in the phylum Basidiomycota were assigned to lichen-associated basidiomycetes, and genes in the Kingdom Bacteria were assigned to lichen-associated bacteria.

Functional annotation of transcripts was performed by using predicted coding regions and blastp searching against the Uniprot database (release 2023_01) with an *E* value threshold of 0.001. Gene functions were inferred by assigning the best *E* value Viridiplantae match for the lichen-forming algae assigned genes, Ascomycete match for the lichen-forming fungus assigned genes, Basidiomycete match for the lichen-associated basidiomycete genes, and Bacteria for the lichen-associated bacteria genes.

Before modeling, only genes that had measurable expression values in all five dry, water vapor, or liquid water replicates were retained. Genes were separated by taxonomy to model the component organisms of the *E. mesomorpha* symbiosis separately. All genes with a best BLAST search *E* value in the class Trebouxiophyceae were filtered and assigned to the lichen-forming alga transcriptome (18,226 genes). All genes with a best BLAST search *E* value in the class Lecanoromycete were filtered and assigned to the lichen-forming fungus transcriptome (6177 genes). All genes with a best BLAST search *E* value in the phylum Basidiomycota were filtered and assigned to the lichen-associated basidiomycete transcriptome (2151). All genes with a best BLAST search *E* value in the kingdom Bacteria were filtered and assigned to the lichen-associated bacteria transcriptome (11,942). To ensure that no genes were erroneously assigned to the lichen-forming fungus (e.g., other ascomycetes living in/on the *E. mesomorpha* lichen), assigned lichen-forming fungus isoforms were BLAST searched against the *Evernia prunastri* genome (*40*). Any genes for which there was no BLAST match were removed from the lichen-forming fungus transcriptome (387). The final filtering step removed genes with a raw total expression value less than 100 summed across all samples, leaving 2424, 3217, 1874, and 5724 genes in the lichen-forming alga and fungus and lichen-associated basidiomycete and bacteria transcriptomes respectively. Differential expression statistics were performed in edgeR (*41*). After prefiltering, expression values were first normalized using edgeR’s trimmed mean of *M* values approach and subsequently fitted with a generalized linear model to account for the three treatment conditions. To test for differential expression, quasi-likelihood *F* tests were performed for each pairwise comparison, and any gene with a false discovery rate < 0.05 and a log-fold change >|1| was considered differentially expressed between the compared conditions.

GO enrichment analysis was performed using the topGO package (*42*). Because no curated databases of GO terms exist for the component organisms of the *E. mesomorpha* symbiosis, custom annotation databases were created from all genes associated with the lichen-forming fungus, alga, and lichen-associated bacteria. GO terms were retrieved by mapping gene identities to GO terms using UniProt’s mapping tool (www.uniprot.org/id-mapping). GO data objects were created with DEGs and tested for enrichment using both the “classic” and “weight01” algorithms with Fisher’s exact test.

### Cross-species comparisons

Lichen specimens representing a wide phylogenetic and morphological range were collected from Minnesota, North Carolina, Mississippi, and California (table S5). Samples were kept frozen (MN material) or air dried and processed within 3 weeks of collection (NC and MS material). A total of 249 thalli within 42 lichen-forming fungi species were measured (table S6).

Hydration conditions and gas exchange measurement conditions were the same as with *E. mesomorpha* (see above). Larger thalli (macrolichens and microlichens attached to larger rocks) were measured using the Bryophyte Chamber (6800-24, internal volume of 193 cm^3^) and smaller thalli (mostly microlichens) were measured using the Aquatic Chamber (6800-18, internal volume of 20 cm^3^). To reduce rates of water loss during measurement (and thus maintain constant hydration levels over the course of measurement periods) with large thalli, low temperature and high humidity conditions were applied: 6°C, RH target 95% (realized RH ~91%), 500 μmol/s flow rate, and reference CO_2_ of 410 ppm. For light measurements, the head light was set at a PAR of 800 μmol/m^−2^ s^−1^ and the color ratio of r90b10. With the smaller thalli, measurements were conducted at room temperature ~26°C in the chamber, RH target 95% (realized RH ~91%), 500 μmol/s flow rate, and reference CO_2_ of 400 ppm. For light measurements, the head light was set at a PAR of 500 μmol m^−2^ s^−1^ and the color ratio of r90b10 for all microlichen specimens, with additional light response curves conducted for at least one specimen per species. When *A*_sat_ exceeded a PAR of 500 μmol m^−2^ s^−1^ (one species, *Roccella*), an additional measurement at saturating light levels (1000 μmol m^−2^ s^−1^) was made. Gas exchange measurements were recorded following stabilization of readings for at least 2 min (typical time, 3 to 5 min), at which point 10 replicate measurements at 1- to 2-s intervals were logged to obtain a mean value. Dark measurements always preceded light measurements.

The carbon balance asymmetry was calculated using the following formula: *A*_prop_ − *R*_prop_, where *A*_prop_ is the proportion of gross assimilation in vapor versus liquid *A*_prop_ = (*A*_net,vapor_ − *R*_vapor_/*A*_net,liquid_ − *R*_liquid_) and *R*_prop_ is the proportion of respiration in vapor versus liquid (*R*_prop_ = *R*_vapor_/*R*_liquid_). We used the average of all specimens tested for each genus to generate the index for the genera shown in Fig. 3.

The cladogram (Fig. 3) was built to represent lineages of fungi and was generated based on (*23*, *24*) for updated phylogenetic positions. Branch length was attributed considering taxonomic hierarchy (class, order, and family) and cladogram visualization was made using the “ggtree” package in R (*43*, *44*). Lichen-forming algal genera were assigned based on (*45*). CCMs of the lichen-forming algae were based on what is known in the literature for the species or genera tested in this study (*25*). For details of all the functional traits shown in Fig. 3, see table S6.

## Supplementary Materials

### This PDF file includes:

Figs. S1 to S4 Tables for S3 to S5 Legends for tables S1, S2, and S6 References
