# Supplementary material for: Symbionts out of sync: Decoupled physiological responses are widespread and ecologically important in lichen associations
Source: Sci Adv. 2024 Jun 14;10(24):eado2783. doi: 10.1126/sciadv.ado2783 (PMC11177896; doi:10.1126/sciadv.ado2783)
Supplement: Supplementary file 1 — Figs. S1 to S4 Tables for S3 to S5 Legends for tables S1, S2, and S6 References [file sciadv.ado2783_sm.pdf]

Science Advances
AAAS

Supplementary Materials for

Symbionts out of sync: Decoupled physiological responses are widespread and ecologically important in lichen associations

Abigail R. Meyer *et al.*

Corresponding author: Daniel E. Stanton, stan0477@umn.edu

Sci. Adv. **10**, eado2783 (2024)
DOI: 10.1126/sciadv.ado2783

The PDF file includes:

Figs. S1 to S4
Tables for S3 to S5
Legends for tables S1, S2, and S6
References

Other Supplementary Material for this manuscript includes the following:

Tables S1, S2, and S6

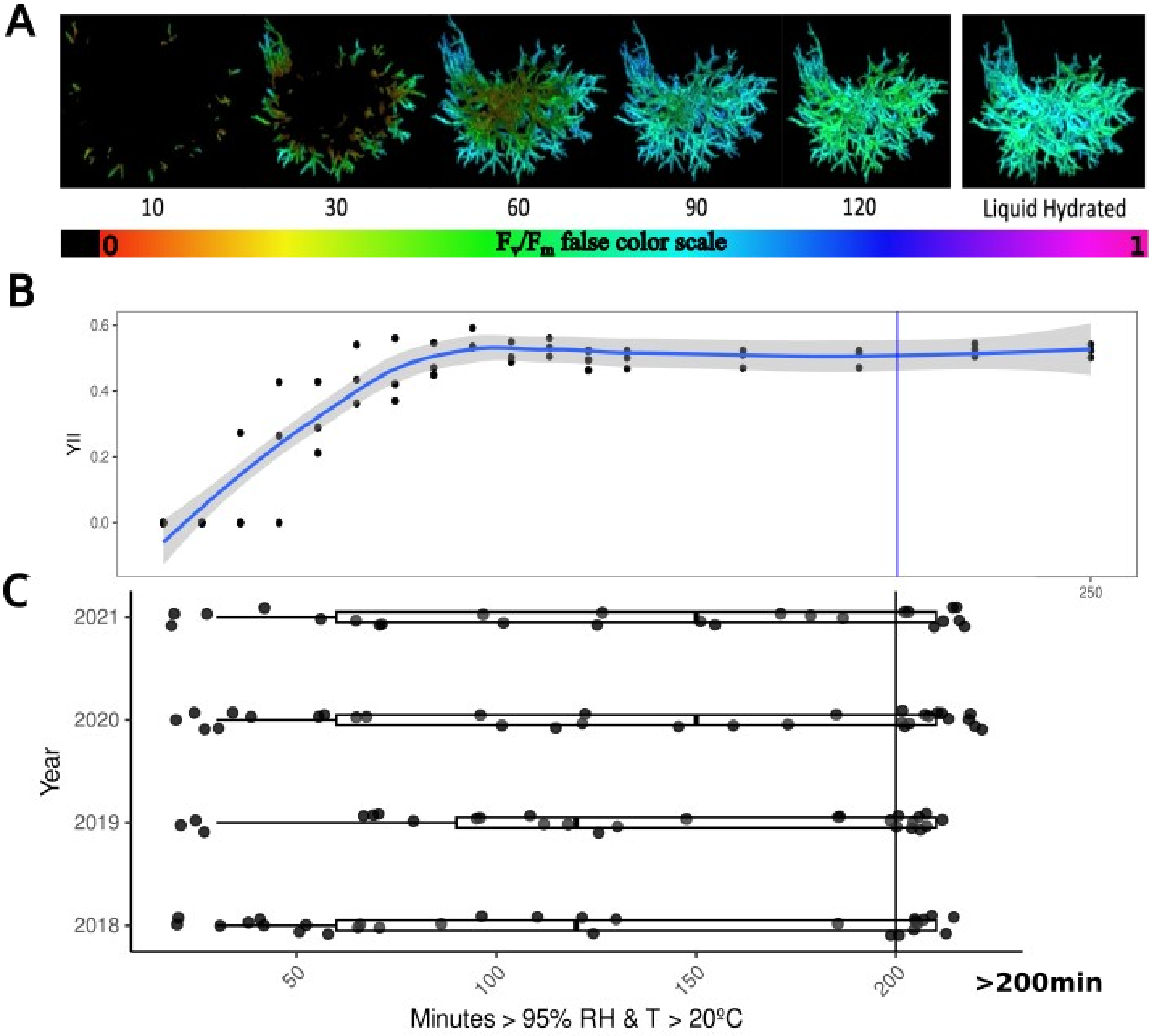

Fig. S1. A-Rapid activation of photosynthetic activity in *Evernia mesomorpha* during exposure to high-humidity conditions and under optimal liquid hydration, as revealed by chlorophyll fluorescence (F_v/F_m). False color scale ranges from 0 (black) to 1 (magenta). B- Rapid activation of Photosystem II Yield in the same thallus of *E. mesomorpha* during hydration under high-humidity conditions, showing rapid activation within less than 200 minutes C- Periods of high humidity (daily hours > 95% RH) and temperature (>20ºC) at a representative site (Marcell Experimental Forest) within the natural range of *E. mesomorpha*. Periods of sufficient duration for full chlorophyll activation at high temperature are consistently frequent across 4 years of records.

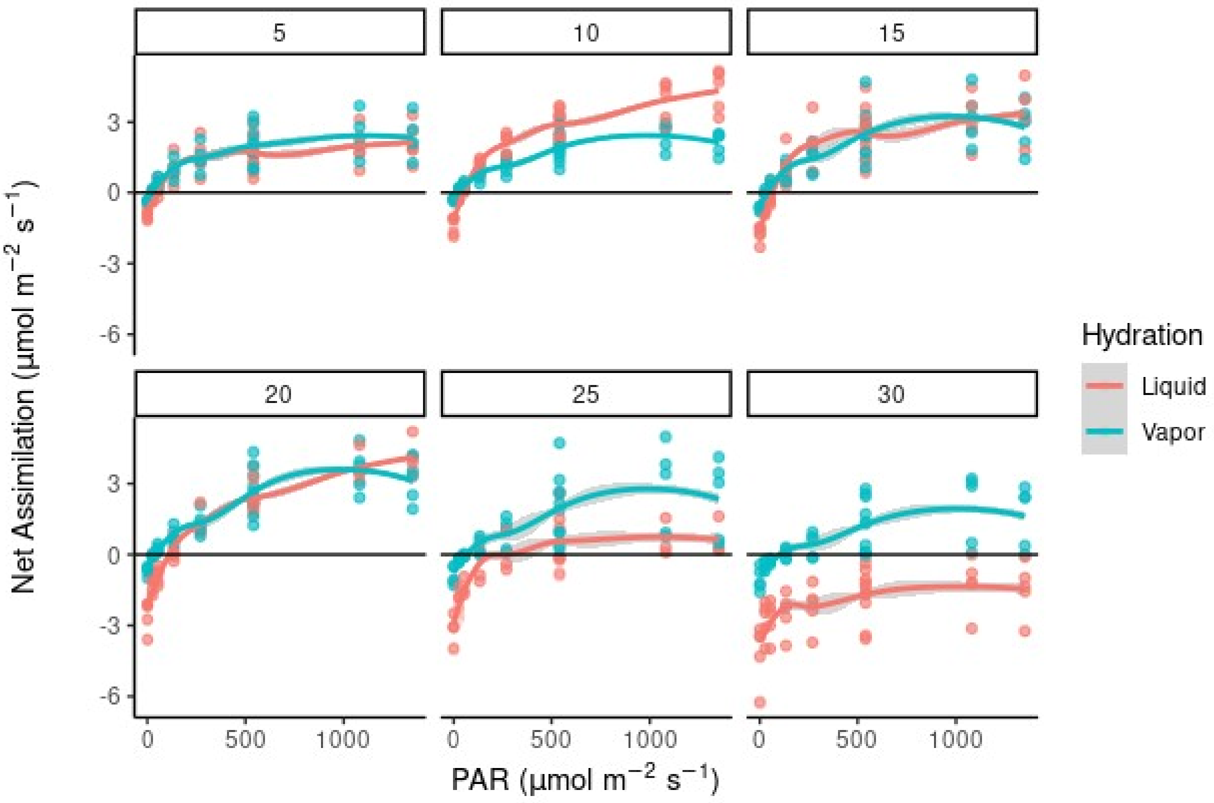

Fig. S2. Light response curves for vapor (blue) and liquid (red) hydrated thalli of *Evernia mesomorpha* at temperatures from 5ºC to 30ºC. Points show mean assimilation values at each light x temperaturexhydration combination for 5 replicate thalli, lines show the smoothed spline of light response for each temperature x hydration combination. At 25ºC, positive instantaneous carbon gain during liquid hydration is only possible at high light (>500 µmol m^{-2} s^{-1}) and at 30ºC liquid-hydrated thalli were unable to achieve positive carbon balance at any light level.

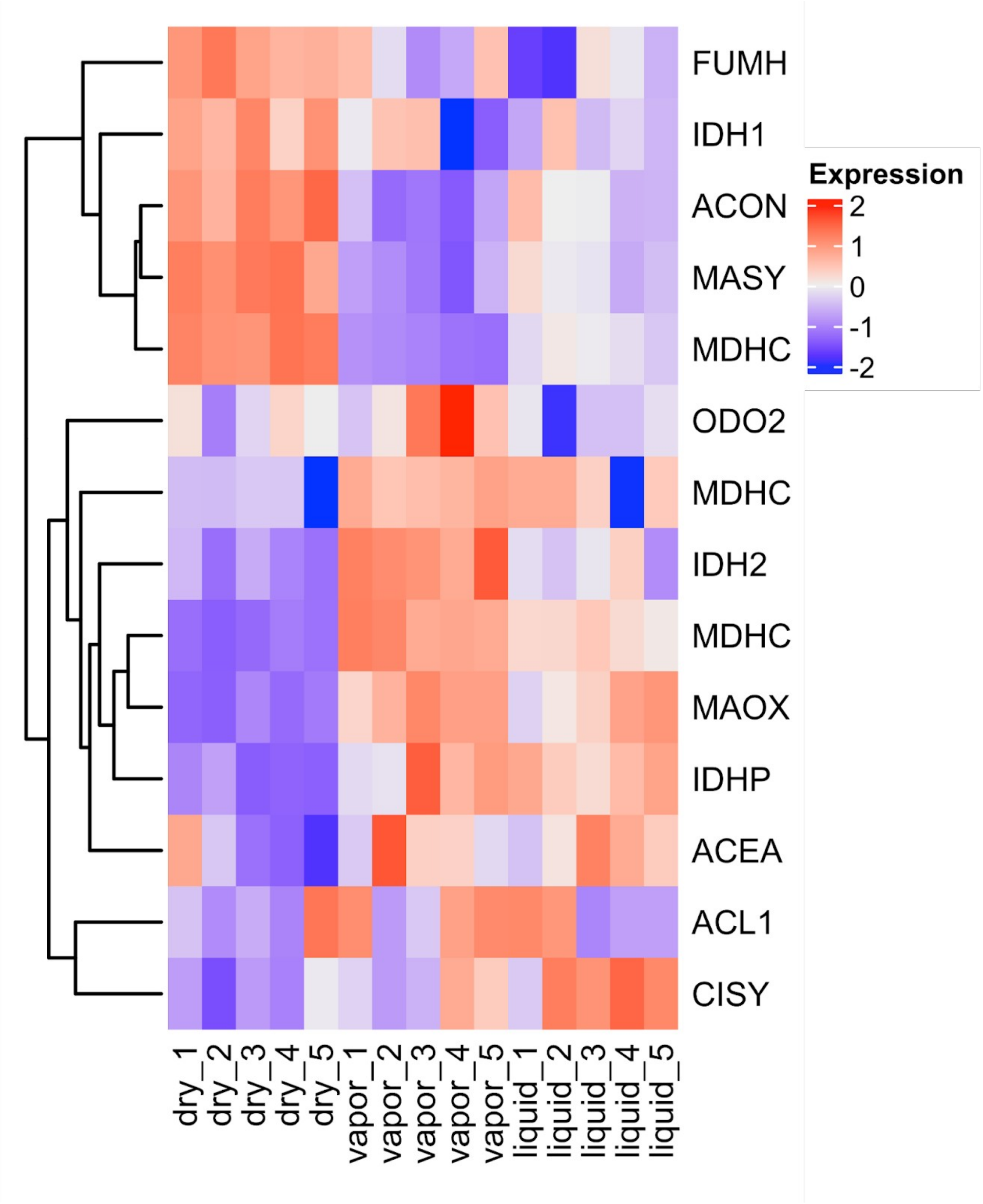

Fig. S3. Heatmap of hierarchically clustered TCA cycle genes (GO:0045239) Full gene names are as follows: FUMH: Fumarate hydratase, IDH1: Isocitrate dehydrogenase [NAD(+)] 1, ACON: Aconitate hydratase, MASY: Malate synthase, MDHC: Malate dehydrogenase,IDH2: Isocitrate dehydrogenase [NAD] subunit 2, MAOX: NAD-dependent malic enzyme, IDHP: Isocitrate dehydrogenase [NADP], ACEA: Isocitrate lyase, ACL1: Probable ATP-citrate synthase subunit 1, CISY: Citrate synthase

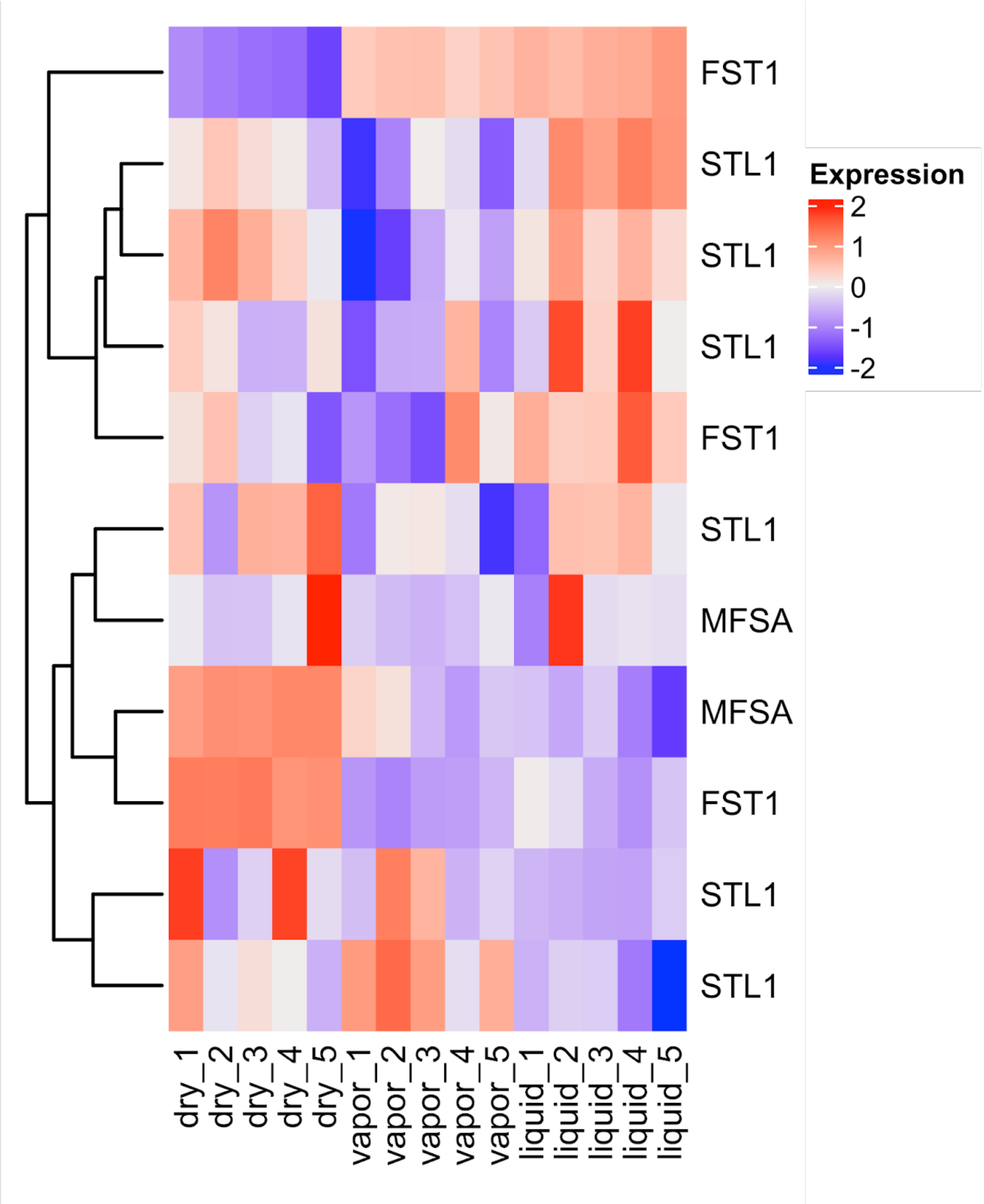

Fig. S4. Heatmap of hierarchically clustered polyol transmembrane transport genes (GO:0015791). Full gene names are as follows: FST1: Myo-inositol transporter, STL1: Sugar transporter, MFSA: Major facilitator superfamily transporter.

File: TableS1_topGO_lichen-forming-fungus.xlsx

Table S1. Summary of Differentially Expressed Genes (DEGs) from the lichen-forming fungus in water vapor vs liquid water hydration conditions. Ten most significantly enriched GO terms in water vapor vs. liquid water lichen-forming fungus DEGs, ranked by the topGO “weight01” algorithm.

File: TableS2_topGO_lichen-forming-bacteria.xlsx

Table S2. Summary of Differentially Expressed Genes (DEGs) from lichen-forming bacteria in water vapor vs liquid water hydration conditions. Ten most significantly enriched GO terms in water vapor vs. liquid water lichen-associated bacteria DEGs, ranked by the topGO "weight01" algorithm.

	Raw Reads	Filtered Reads
Dry_1	53,259,118	30,126,895
Dry_2	57,420,987	35,562,930
Dry_3	60,650,122	35,336,225
Dry_4	53,165,775	28,321,468
Dry_5	52,576,837	27,384,580
Liquid_1	68,358,485	35,533,548
Liquid_2	50,105,931	25,965,300
Liquid_3	59,761,933	31,721,137
Liquid_4	66,751,315	34,952,589
Liquid_5	55,422,892	28,623,557
Vapor_1	74,014,428	40,377,421
Vapor_2	56,074,720	29,005,864
Vapor_3	58,035,899	29,984,164
Vapor_4	57,635,067	31,078,634
Vapor_5	55,531,483	30,186,286

Table S3. Raw and filtered read counts from transcriptome analyses of *E. mesomorpha* thalli under different hydration treatments

**Counts of transcripts**	
Total trinity 'genes':	917783
Total trinity transcripts:	1648858
Percent GC:	51.38
**Statistics based on all transcript contigs**	
Contig N10:	23789
Contig N20:	17908
Contig N30:	14008
Contig N40:	11204
Contig N50	8538
Median contig length:	405
Average contig:	1873.93
Total assembled bases	3089849594
**Statistics based on the longest isoform per trinity 'gene'**	
Contig N10:	4355
Contig N20:	1613
Contig N30:	870
Contig N40:	586
Contig N50	446
Median contig length:	299
Average contig:	447.64
Total assembled bases:	410836752

Table S4. Assembly statistics

Order	Taxon	Source	Asymmetry	Reference	Notes
Acarosporales	*Acarospora conafii*	Chile	none	46	
Acarosporales	*Acarospora gwynnii*	Antarctica	none	47	
Arthoniales	*Roccella fucoides*	Italy	positive	14	
Caliciales	*Buellia frigida*	Antarctica	positive	56	
Caliciales	*Heterodermia spinulosa*	Chile	positive	48	
Hymeneliales	*Hymenelia coerulea*	Alps	positive	12	
Hymeneliales	*Hymenelia prevostii*	Alps	positive	12	
Lecanorales	*Cetraria crispa*	Germany	positive	49	
Lecanorales	*Cetraria islandia*	Germany	none	49	
Lecanorales	*Cetraria islandica*	Italy	positive	49	
Lecanorales	*Cladonia alcicornis*	Germany	positive	49	
Lecanorales	*Cladonia furcata*	Germany	negative	49	
Lecanorales	*Cladonia gracilis*	Germany	positive	49	
Lecanorales	*Cladonia impexa*	Germany	positive	49	
Lecanorales	*Cladonia mitis*	Italy	likely-not shown	15	full data not shown
Lecanorales	*Cladonia portentosa*	Germany	likely-not shown	15	full data not shown
Lecanorales	*Cladonia rangiferina*	Germany	negative	49	
Lecanorales	*Cladonia rangiferina*	Italy	likely-not shown	15	full data not shown
Lecanorales	*Cladonia sylvatica*	Germany	positive	49	
Lecanorales	*Cladonia uncialis*	Germany	positive	49	
Lecanorales	*Cornicularia aculeata*	Germany	positive	49	
Lecanorales	*Cornicularia muricata*	Germany	positive	49	
Lecanorales	*Evernia divaricata*	Germany	positive	50	
Lecanorales	*Evernia prunastri*	Germany	positive	50	
Lecanorales	*Everniopsis trulla*	Chile	positive	48	
Lecanorales	*Everniopsis trulla*	Chile		15	data from previous study
Lecanorales	*Everniopsis trulla*	Chile	positive	46	
Lecanorales	*Heterodermia spinulosa*	Chile		15	data from previous study
Lecanorales	*Lecanora melanophthalma*	Antarctica	negative	56	
Lecanorales	*Neuropogon acromelanus*	Antarctica	positive	56	
Lecanorales	*Parmelia caperata*	Portugal	likely-not shown	15	full data not shown
Lecanorales	*Parmelia caperata*	Portugal	likely-not shown	15	full data not shown
Lecanorales	*Parmelia hypoleucina*	Portugal	likely-not shown	15	full data not shown
Lecanorales	*Parmelia perlata*	Portugal	likely-not shown	15	full data not

					shown
Lecanorales	*Pseudephebe minuscula*	Italy	likely-not shown	15	full data not shown
Lecanorales	*Ramalina cactacearum*	Chile	positive	48	
Lecanorales	*Ramalina cactacearum*	Chile		15	data from previous study
Lecanorales	*Ramalina capitata*	Spain	positive	51	
Lecanorales	*Ramalina farinacea*	Germany	positive	50	
Lecanorales	*Ramalina fenestrata*	Portugal	likely-not shown	15	full data not shown
Lecanorales	*Ramalina maciformis*	Israel	positive	52	
Lecanorales	*Ramalina maciformis*	Israel		15	data from previous study
Lecanorales	*Ramalina menziesii*	California	positive	14	
Lecanorales	*Ramalina thrausta*	Germany	positive	50	
Lecanorales	*Ramalina thrausta*	Chile	positive	46	
Lecanorales	*Ramalina vizzavonensis*	Portugal	positive	15	
Lecanorales	*Ramalina vizzavonensis*	Portugal	likely-not shown	15	full data not shown
Lecanorales	*Stereocaulon vesuvianum*	Portugal	likely-not shown	15	full data not shown
Lecanorales	*Usnea fasciata*	South Shetland Islands	likely-not shown	15	full data not shown
Lecanorales	*Usnea lacerata*	Chile	positive	48	
Lecanorales	*Usnea lacerata*	Chile		15	data from previous study
Lecanorales	*Usnea subpectinata*	Portugal	likely-not shown	15	full data not shown
Lecanorales	*Usnea subscabrosa*	Portugal	likely-not shown	15	full data not shown
Lecanorales	*Usnea sulphurea*	Antarctica	positive	53	
Lichinales	*Gonohymenia crebilifera*	Arizona	none	14	
Lichinales	*Lempholemma chalazanum*	Germany	negative	14	
Lichinales	*Thyrea rotundata*	South Africa	none	14	
Peltigerales	*Collema auriculatum*	Germany	negative	14	
Peltigerales	*Collema crispum*	England	negative	15	
Peltigerales	*Collema cristatum*	Germany	negative	15	
Peltigerales	*Collema cristatum*	Austria	negative	14	
Peltigerales	*Collema occultatum*	Alps	negative	14	
Peltigerales	*Leptogium brebissonii*	Portugal	negative	15	
Peltigerales	*Leptogium lichenoides*	Germany	negative	14	

Peltigerales	*Leptogium saturninum*	Alps	negative	14	
Peltigerales	*Lobaria laetivirens*	Portugal	positive	14	
Peltigerales	*Lobaria pulmonaria*	Canary Islands	positive	14	
Peltigerales	*Lobaria scrobiculata*	Alps	negative	14	
Peltigerales	*Nephroma resupinatum*	Alps	none	14	
Peltigerales	*Pannaria conoplea*	Alps	negative	14	
Peltigerales	*Parmeliella plumbea*	Canary Islands	negative	14	
Peltigerales	*Parmeliella triptophylla*	Alps	negative	14	
Peltigerales	*Peltigera leucophlebia*	Alps	positive	14	
Peltigerales	*Peltigera leucophlebia*	Alaska	positive	14	
Peltigerales	*Peltigera leucophlebia*	Alps	positive	14	
Peltigerales	*Peltigera neckeri*	Alps	negative	14	
Peltigerales	*Peltigera polydactyla*	Germany	none	14	
Peltigerales	*Peltigera praetextata*	Alps	negative	14	
Peltigerales	*Peltigera rufescens*	Germany	none	14	
Peltigerales	*Placynthium nigrum*	Germany	none	14	
Pertusariales	*Thamnolia vermicularis*	Italy	likely-not shown	15	full data not shown
Teloschistales	*Caloplaca regalis*	King George Island	likely-not shown	15	full data not shown
Teloschistales	*Teloschistes capensis*	Namibia	positive	54	
Teloschistales	*Teloschistes lacunosus*	Spain	none	55	
Teloschistales	*Xanthoria mawsoni*	Antarctica	positive	56	
Umbilicariales	*Lasallia pustulata*	Portugal	likely-not shown	15	full data not shown
Umbilicariales	*Umbilicaria antarctica*	South Shetland Islands	likely-not shown	15	full data not shown
Umbilicariales	*Umbilicaria crustulosa*	Alps	likely-not shown	15	full data not shown
Umbilicariales	*Umbilicaria cylindrica*	Italy	positive	15	
Umbilicariales	*Umbilicaria deusta*	Italy	positive	15	
Verrucariales	*Placidium* sp.	Chile	none	46	

Table S5. Prior reports of vapor-phase gas-exchange in lichens. Compilation of previous comparisons of vapor and liquid hydrated gas-exchange in lichens, and whether the data show asymmetry. In some cases data for that specific taxon was not shown, but may be inferred from the text. Taxon names are as reported in the original, and organized by taxonomic order following current classifications (23).

File: TableS6_Figure3Data.xlsx

Table S6. Traits and collection locations of the lichens surveyed for physiological asymmetry. Primary fungal species and algal genus for all taxa included in the broad survey across lichen lineages, as well as associated traits. Traits include carbon balance asymmetry (see supplementary data for raw data), growth form, ecological substrate, presence and type of carbon concentrating mechanism and source location (US state, Latitude, Longitude).

REFERENCES AND NOTES

1. D. C. Smith, A. E. Douglas, *The Biology of Symbiosis.* (Edward Arnold (Publishers) Ltd., London, 1987).

2. J. L. Sachs, E. L. Simms, Pathways to mutualism breakdown. *Trends Ecol. Evol.* **21**, 585–592 (2006).

3. E. T. Kiers, T. M. Palmer, A. R. Ives, J. F. Bruno, J. L. Bronstein, Mutualisms in a changing world: An evolutionary perspective. *Ecol. Lett.* **13**, 1459–1474 (2010).

4. N. Rädecker, C. Pogoreutz, H. M. Gegner, A. Cárdenas, F. Roth, J. Bougoure, P. Guagliardo, C. Wild, M. Pernice, J.-B. Raina, A. Meibom, C. R. Voolstra, Heat stress destabilizes symbiotic nutrient cycling in corals. *Proc. Natl. Acad. Sci. U.S.A.* **118**, e2022653118 (2021).

5. A. Frank, Über die biologischen Vehältnisse des Thallus einiger Krustenflechten. *Beiträge zur Biologie Pflanzen* **2**, 123–200 (1876).

6. G. Tagirdzhanova, P. Saary, J. P. Tingley, D. Díaz-Escandón, D. W. Abbott, R. D. Finn, T. Spribille, Predicted input of uncultured fungal symbionts to a lichen symbiosis from metagenome-assembled genomes. *Genome Biol. Evol.* **13**, evab047 (2021).

7. T. Spribille, P. Resl, D. E. Stanton, G. Tagirdzhanova, Evolutionary biology of lichen symbioses. *New Phytol.* **234**, 1566–1582 (2022).

8. M. Grimm, M. Grube, U. Schiefelbein, D. Zühlke, J. Bernhardt, K. Riedel, The lichens' microbiota, still a mystery? *Front. Microbiol.* **12**, 623839 (2021).

9. G. Tagirdzhanova, P. Saary, E. S. Cameron, A. I. Garber, D. D. Escandón, S. Goyette, V. T. Nogerius, A. Passo, H. Mayrhofer, H. Holien, T. Tønsberg, L. Y. Stein, R. D. Finn, T. Spribille, Evidence for a core set of microbial lichen symbionts from a global survey of metagenomes. *bioRxiv*, https://doi.org/10.1101/2023.02.02.524463 (2023).

10. A. R. Meyer, M. Valentin, L. Liulevicius, T. R. McDonald, M. P. Nelsen, J. Pengra, R. J. Smith, D. Stanton, Climate warming causes photobiont degradation and carbon starvation in a boreal climate sentinel lichen. *Am. J. Bot.* **110**, e16114 (2023).

11. N. H. Phinney, K. A. Solhaug, Y. Gauslaa, Photobiont-dependent humidity threshold for chlorolichen photosystem II activation. *Planta* **250**, 2023–2031 (2019).

12. B. Weber, C. Scherr, H. Reichenberger, B. Büdel, Fast reactivation by high air humidity and photosynthetic performance of alpine lichens growing endolithically in limestone. *Arct. Antarct. Alp. Res.* **39**, 309–317 (2007).

13. N. H. Phinney, K. A. Solhaug, Y. Gauslaa, Rapid resurrection of chlorolichens in humid air: Specific thallus mass drives rehydration and reactivation kinetics. *Environ. Exp. Bot.* **148**, 184–191 (2018).

14. O. L. Lange, E. Kilian, H. Ziegler, Water vapor uptake and photosynthesis of lichens: Performance differences in species with green and blue-green algae as phycobionts. *Oecologia* **71**, 104–110 (1986).

15. O. L. Lange, E. Kilian, Reaktivierung der Photosynthese trockener Flechten durch Wasserdampfaufnahme aus dem Luftraum: Artspezifisch unterschiedliches Verhalten[1] reactivation of photosynthesis of dry lichen thalli through water vapor uptake from air: Species-specific response patterns. *Flora* **176**, 7–23 (1985).

16. D. E. Stanton, A. Ormond, N. M. Koch, C. Colesie, Lichen ecophysiology in a changing climate. *Am. J Bot.* **110** e16131 (2023).

17. O. Johansson, J. Olofsson, R. Giesler, K. Palmqvist, Lichen responses to nitrogen and phosphorus additions can be explained by the different symbiont responses. *New Phytol.* **191**, 795–805 (2011).

18. K. Palmqvist, O. Franklin, T. Näsholm, Symbiosis constraints: Strong mycobiont control limits nutrient response in lichens. *Ecol. Evol.* **7**, 7420–7433 (2017).

19. O. L. Lange, T. G. A. Green, U. Heber, Hydration-dependent photosynthetic production of lichens: What do laboratory studies tell us about field performance? *J. Exp. Bot.* **52**, 2033–2042 (2001).

20. Consortium of Lichen Herbaria Data Portal, *Consortium of Lichen Herbaria* (2023). http://lichenportal.org/portal/index.php.

21. S. E. Fick, R. J. Hijmans, WorldClim 2: New 1-km spatial resolution climate surfaces for global land areas. *Int. J. Climatol.* **37**, 4302–4315 (2017).

22. F. A. Hasby, F. Barbi, S. Manzoni, B. D. Lindahl, Transcriptomic markers of fungal growth, respiration and carbon-use efficiency. *FEMS Microbiol. Lett.* **368**, fnab100 (2021).

23. R. Lücking, B. P. Hodkinson, S. D. Leavitt, The 2016 classification of lichenized fungi in the Ascomycota and Basidiomycota–Approaching one thousand genera. *Bryologist* **119**, 361–416 (2016).

24. D. Díaz-Escandón, G. Tagirdzhanova, D. Vanderpool, C. C. G. Allen, A. Aptroot, O. Češka, D. L. Hawksworth, A. Huereca, K. Knudsen, J. Kocourková, R. Lücking, P. Resl, T. Spribille, Genome-level analyses resolve an ancient lineage of symbiotic ascomycetes. *Curr. Biol.* **32**, 5209–5218.e5 (2022).

25. N. M. Koch, J. C. Lendemer, E. A. Manzitto-Tripp, C. McCain, D. E. Stanton, Carbon-concentrating mechanisms are a key trait in lichen ecology and distribution. *Ecology* **104**, e4011 (2023).

26. N. Chrismas, B. Tindall-Jones, H. Jenkins, J. Harley, K. Bird, M. Cunliffe, Metatranscriptomics reveals diversity of symbiotic interaction and mechanisms of carbon exchange in the marine cyanolichen Lichina pygmaea. *New Phytol.* **241**, 2243–2257 (2024).

27. B. Wilske, J. Burgheimer, A. Karnieli, E. Zaady, M. O. Andreae, D. Yakir, J. Kesselmeier, The CO_2 exchange of biological soil crusts in a semiarid grass-shrubland at the northern transition zone of the Negev desert, Israel. *Biogeosciences* **5**, 1411–1423 (2008).

28. S. C. Reed, K. K. Coe, J. P. Sparks, D. C. Housman, T. J. Zelikova, J. Belnap, Changes to dryland rainfall result in rapid moss mortality and altered soil fertility. *Nat. Clim. Change* **2**, 752–755 (2012).

29. M. Lakatos, U. Rascher, B. Büdel, Functional characteristics of corticolous lichens in the understory of a tropical lowland rain forest. *New Phytol.* **172**, 679–695 (2006).

30. M. D. Piercey-Normore, The lichen-forming ascomycete Evernia mesomorpha associates with multiple genotypes of Trebouxia jamesii. *New Phytol.* **169**, 331–344 (2006).

31. L. M. Casano, E. M. del Campo, F. J. García-Breijo, J. Reig-Armiñana, F. Gasulla, A. del Hoyo, A. Guéra, E. Barreno, Two Trebouxia algae with different physiological performances are ever-present in lichen thalli of Ramalina farinacea. Coexistence versus competition? *Environ. Microbiol.* **13**, 806–818 (2011).

32. A. R. Cruz, G. Davidowitz, C. M. Moore, J. L. Bronstein, Mutualisms in a warming world. *Ecol. Lett.* **26**, 1432–1451 (2023).

33. R. J. Hijmans, *Terra: Spatial Data Analysis* (2023; https://CRAN.R-project.org/package=terra).

34. P. J. Hanson, J. S. Riggs, W. R. Nettles, M. B. Krassovski, L. A. Hook, *SPRUCE Whole Ecosystems Warming (WEW) Environmental Data Beginning August 2015*, Oak Ridge National Laboratory, TES SFA, U.S. Department of Energy, Oak Ridge, Tennessee, U.S.A. (2016); https://doi.org/10.3334/CDIAC/spruce.032.

35. D. Armaleo, O. Müller, F. Lutzoni, Ó. S. Andrésson, G. Blanc, H. B. Bode, F. R. Collart, F. Dal Grande, F. Dietrich, I. V. Grigoriev, S. Joneson, A. Kuo, P. E. Larsen, J. M. Logsdon, D. Lopez, F. Martin, S. P. May, T. R. McDonald, S. S. Merchant, V. Miao, E. Morin, R. Oono, M. Pellegrini, N. Rubinstein, M. V. Sanchez-Puerta, E. Savelkoul, I. Schmitt, J. C. Slot, D. Soanes, P. Szövényi, N. J. Talbot, C. Veneault-Fourrey, B. B. Xavier, The lichen symbiosis re-viewed through the genomes of Cladonia grayi and its algal partner Asterochloris glomerata. *BMC Genomics* **20**, 605 (2019).

36. Z.-L. Deng, P. C. Münch, R. Mreches, A. C. McHardy, Rapid and accurate identification of ribosomal RNA sequences via deep learning. *Nucleic Acids Res.* **50**, e60 (2022).

37. M. G. Grabherr, B. J. Haas, M. Yassour, J. Z. Levin, D. A. Thompson, I. Amit, X. Adiconis, L. Fan, R. Raychowdhury, Q. Zeng, Z. Chen, E. Mauceli, N. Hacohen, A. Gnirke, N. Rhind, F. di Palma, B. W. Birren, C. Nusbaum, K. Lindblad-Toh, N. Friedman, A. Regev, Full-length transcriptome assembly from RNA-Seq data without a reference genome. *Nat. Biotechnol.* **29**, 644–652 (2011).

38. T. Spribille, V. Tuovinen, P. Resl, D. Vanderpool, H. Wolinski, M. C. Aime, K. Schneider, E. Stabentheiner, M. Toome-Heller, G. Thor, H. Mayrhofer, H. Johannesson, J. P. McCutcheon, Basidiomycete yeasts in the cortex of ascomycete macrolichens. *Science* **353**, 488–492 (2016).

39. S. Sherrill-Mix, *Taxonomizr: Functions to Work with NCBI Accessions and Taxonomy* (2023; https://CRAN.R-project.org/package=taxonomizr).

40. A. Meiser, J. Otte, I. Schmitt, F. D. Grande, Sequencing genomes from mixed DNA samples—Evaluating the metagenome skimming approach in lichenized fungi. *Sci. Rep.* **7**, 14881 (2017).

41. M. D. Robinson, D. J. McCarthy, G. K. Smyth, edgeR: A Bioconductor package for differential expression analysis of digital gene expression data. *Bioinformatics* **26**, 139–140 (2010).

42. A. Alexa, J. Rahnenfuhrer, *topGO: Enrichment Analysis for Gene Ontology* (2023; https://bioconductor.org/packages/topGO).

43. G. Yu, Using ggtree to visualize data on tree-like structures. *Curr. Protoc. Bioinformatics* **69**, e96 (2020).

44. G. Yu, D. K. Smith, H. Zhu, Y. Guan, T. T.-Y. Lam, GGTREE: An R package for visualization and annotation of phylogenetic trees with their covariates and other associated data. *Methods Eco. Evol.* **8**, 28–36 (2017).

45. W. B. Sanders, H. Masumoto, Lichen algae: The photosynthetic partners in lichen symbioses. *Lichenologist* **53**, 347–393 (2021).

46. P. Jung, D. Emrich, L. Briegel-Williams, M. Schermer, L. Weber, K. Baumann, C. Colesie, P. Clerc, L. W. Lehnert, S. Achilles, J. Bendix, B. Büdel, Ecophysiology and phylogeny of new terricolous and epiphytic chlorolichens in a fog oasis of the Atacama Desert. *Microbiologyopen* **8**, e894 (2019).

47. C. Colesie, T. A. Green, I. Haferkamp, B. Büdel, Habitat stress initiates changes in composition, CO2 gas exchange and C-allocation as life traits in biological soil crusts. *ISME J.* **8**, 2104–2115 (2014).

48. O. L. Lange, J. Redón, Epiphytische Flechten im Bereich einer chilenischen “Nebeloase”(Fray Jorge). II Ökophysiologische Charakterisierung von CO 2 -Gaswechsel und Wasserhaushalt. *Flora* **174**, 245–284 (1983).

49. R. Büttner, Untersuchungen zur Ökologie und Physiologie des Gasstoffwechsels bei einigen Strauchflechten[1] investigations of ecology and physiology of gas exchange in several fruticose lichens. *Flora* **160**, 72–99 (1971).

50. A. Bertsch, Über den CO2-Gaswechsel einiger Flechten nach Wasserdampfaufnahme. *Planta* **68**, 157–166 (1966).

51. A. Pintado, L. G. Sancho, Ecological significance of net photosynthesis activation by water vapour uptake in Ramalina capitata from rain-protected habitats in central Spain. *Lichenologist* **34**, 403–413 (2002).

52. O. L. Lange, A. Bertsch, Photosynthese der Wüstenflechte *Ramalina maciformis* nach Wasserdampfaufnahme aus dem Luftraum. *Naturwissenschaften* **52**, 215–216 (1965).

53. L. Kappen, Ecology and physiology of the Antarctic fruticose lichen *Usnea sulphurea* (Koenig) Th. Fries. *Polar Biol.* **1**, 249–255 (1983).

54. O. L. Lange, A. Meyer, H. Zellner, I. Ullmann, D. C. J. Wessels, Eight days in the life of a desert lichen: Water relations and photosynthesis of *Teloschistes capensis* in the coastal fog zone of the Namib Desert. *Madoqua* **174**, 17–30 (1990).

55. R. del Prado, L. G. Sancho, Dew as a key factor for the distribution pattern of the lichen species *Teloschistes lacunosus* in the Tabernas Desert (Spain). *Flora* **202**, 417–428 (2007).

56. O. L. Lange, L. Kappen, *Photosynthesis of Lichens from Antarctica* in Antarctic Terrestrial Biology (American Geophysical Union (AGU), 1972, pp. 83–95.
